# Leg edema with (S)-amlodipine *vs* conventional amlodipine given in triple therapy for hypertension: a randomized double blind controlled clinical trial

**DOI:** 10.1186/s12872-016-0350-z

**Published:** 2016-09-01

**Authors:** Priyadarshani Galappatthy, Yasindu C. Waniganayake, Mohomad I. M. Sabeer, Thusitha J. Wijethunga, Gamini K. S. Galappatthy, Ruvan AI Ekanayaka

**Affiliations:** 1 Department of Pharmacology and Pharmacy, Faculty of Medicine, University of Colombo, PO Box 271, Kynsey Road, Colombo, Sri Lanka; 2 Institute of Cardiology, National Hospital of Sri Lanka, Colombo, Sri Lanka

**Keywords:** (S)-Amlodipine, Conventional racemic amlodipine, Calcium channel blocker, Leg edema, Hypertension, Randomized controlled clinical trial

## Abstract

**Background:**

Leg edema is a common adverse effect of dihydropyridine Calcium Channel Blockers (CCB) that may need dose reduction or drug withdrawal, adversely affecting the antihypertensive efficacy. Leg edema is reported to occur less often with (S)-amlodipine compared to conventional racemic amlodipine. We aimed to find the incidence of leg edema as a primary outcome and antihypertensive efficacy with (S)-amlodipine compared to conventional amlodipine.

**Methods:**

This prospective, double-blind, controlled clinical trial randomized 172 hypertensive patients, not controlled on beta-blockers (BB) and angiotensin converting enzyme inhibitors/angiotensin receptor blockers (ACEI/ARB), to either conventional amlodipine (5–10 mg; *n =* 86) or (S)-amlodipine (2.5–5 mg; *n =* 86), while continuing their previous anti-hypertensive medications. Sample was sufficient to find a difference in edema between the interventions with 80 % power at 5 % significance level. Intension to treat analysis (ITT) for safety data and per protocol analysis for efficacy data was performed. Fischer’s exact test was applied to observe difference between responder rates and proportions of subjects having peripheral edema in the two groups. Pitting edema test scores were compared using Mann–Whitney test.

**Results:**

Altogether 146 patients (amlodipine, *n =* 76 and (S)-amlodipine, *n =* 70) completed 120 days treatment. Demographic variables and treatment adherence were comparable in the two groups. Incidence of new edema after randomization was 31.40 % in test group and 46.51 % in control group [*p* = 0.03; absolute risk reduction (ARR) = 15.1 %; Number Needed to Treat (NNT) = 7, ITT analysis]. Pitting edema score and patient rated edema score increased significantly in the control compared to test group (*p* = 0.038 and 0.036 respectively) after treatment period. Edema scores increased significantly in the control group from baseline (*p* < 0.0001). Responders in blood pressure were 98.57 % in test and 98.68 % in control group. Most common adverse events (AE) were pitting edema and increased urinary frequency. Incidence of all AEs other than edema was similar in both groups. Two serious AEs occurred unrelated to therapy. Biochemical and ECG parameters in the two groups were comparable.

**Conclusions:**

In hypertensive patients not controlled on prior BB and ACEI/ARB therapy, addition of (S)-amlodipine besylate at half the dose of conventional amlodipine provides better tolerability with reduced incidence of peripheral edema, and equal antihypertensive efficacy compared to amlodipine given at usual doses.

**Trial registration:**

Sri Lanka Clinical Trials registry: www.slctr.lk, SLCTR/2013/006

## Background

Amlodipine is a calcium channel blocker (CCB) of the third generation dihydropyridine CCB group, used as monotherapy or in combination, for treatment of hypertension and angina [1–4]. Peripheral edema, particularly of the lower limbs, is a common adverse effect of dihydropyridine CCB. This might lead to dose reduction or drug withdrawal, adversely affecting the antihypertensive efficacy [5–7]. The degree of peripheral edema that occurs with CCB treatment is dependent on the dose and the drug used [8–10].

Reported rates of peripheral edema with CCBs varies widely because of the drug and dose-dependent nature, ranging from 5 to even 70 % with high doses [5, 9–12]. The incidence of edema reported in the literature can also be dependent on the method of edema assessment in the clinical trials [13, 14].

Racemic or conventional amlodipine contains (R)-and (S)-amlodipine isomers in a 1:1 ratio, but (S)-amlodipine has been shown to be the only active isomer of amlodipine which has calcium channel blocking activity, providing all the therapeutic effects [15]. Therefore, an amlodipine formulation composed of only (S)-amlodipine has been developed [16]. (S)-amlodipine is in use in many Asian, African, Latin American and Commonwealth of Independent States (CIS) countries. The standard dose of (S)-amlodipine recommended is half that of racemic amlodipine.

Although few published studies including a meta analysis [17] indicated that (S)-amlodipine and racemic amlodipine show similar efficacy and safety [18, 19], data from a post marketing study involving over 5000 patients has shown that the incidence of leg edema with (S)-amlodipine is as low as 1.56 % overall [20]. Also, (S)-amlodipine has been recommended as an ideal therapy for switching from conventional racemic amlodipine for patients developing peripheral edema [21].

Although several clinical trials comparing amlodipine and (S)-amlodipine have been done, no adequately powered, properly conducted, randomised controlled clinical trial has compared the incidence of leg edema as a primary outcome between the two drugs. The influence of (S)-amlodipine compared to racemic amlodipine on edema incidence in patients on CCB, beta blocker (BB) and Angiotensin Converting Enzyme Inhibitor (ACEI)/Angiotensin Receptor Blocker (ARB) triple therapy has also not been assessed in a clinical trial.

Therefore the objective of this study was to assess and compare the antihypertensive efficacy and incidence of leg edema as a primary outcome, between (S)-amlodipine and racemic amlodipine in the majority of patients with essential hypertension, not controlled on a BB and an ACEI/ARB in a tertiary care referral centre.

## Methods

### Trial design

This study was a prospective, randomized, double-blind, double-dummy, comparative, parallel-group clinical trial with an allocation ratio of 1:1. The Ethics Review Committee, Faculty of Medicine, University of Colombo (EC-10-014) approved the study protocol. The Cosmetics Devices and Drugs Authority (CDDA) of Ministry of Health, Sri Lanka gave the regulatory approval for the conduct of the trial and for import of study medication, as (S)-amlodipine is not a registered product in Sri Lanka. The study was registered at the Clinical Trials Registry of the Sri Lanka Medical Association, a publicly accessed clinical trials registry recognised by the WHO, (www.slctr.lk, SLCTR/2013/006). The study was conducted in accordance with Good Clinical Practice (GCP) guidelines.

### Participants

Subjects visiting the Out Patient Department (OPD) for treatment of hypertension at Institute of Cardiology National Hospital of Sri Lanka, Colombo were enrolled in the study. Male and female subjects with essential hypertension taking a BB and ACEI/ARB, not responding to therapy (BP ≥140/90 mmHg) were included. All subjects provided written informed consent.

Inclusion criteria were subjects between 30–64 years of age who had essential hypertension and on stable dose of BB and ACEI/ARB for at least 4 weeks, having either systolic BP (SBP) ≥140 mmHg or diastolic BP (DBP) ≥90 mmHg, or both, in sitting position.

Subjects sensitive to amlodipine, CCB or to any of the ingredients of the test/reference product, taking CCB or any antihypertensive therapy other than beta blocker and ACEI/ARB, those with history of secondary, resistant or malignant hypertension, myocardial infarction, percutaneous trans coronary angiogram (PTCA), coronary artery bypass grafting (CABG), cerebrovascular accident, or transient ischemic attack (TIA) in last 6 months were excluded. Patients with clinical diagnosis of congestive cardiac failure, cardiac arrhythmias, any other abnormality on ECG, known significant respiratory, liver, kidney, neurological diseases, coagulation disorders, metabolic or endocrinal disorder (except type 2 diabetes mellitus), uncontrolled diabetes mellitus or type 2 diabetes mellitus taking insulin or oral hypoglycemic drugs other than metformin or sulfonylurea and more than one sulfonylurea were also excluded. Pregnant and lactating women or the women in the reproductive age group not practicing the effective means of contraception, patients with known alcohol or drug abuse and any condition that, in the opinion of the investigator, does not justify inclusion in the study were also excluded.

### Interventions

The test medication used was tablets containing (S)-amlodipine besylate 5 mg (high dose) and 2.5 mg (low dose). The reference medication was amlodipine besylate 10 mg (high dose) and 5 mg (low dose). The starting dose of conventional amlodipine was the usual maintenance dose required by patients of 10 mg daily and corresponding (S)-amlodipine dose of 5 mg daily. These starting doses were down titrated to 5 mg conventional amlodipine and 2.5 mg (S)-amlodipine by the investigators if BP was considered too low (<115/75 mmHg) or patients was unable to tolerate the dose or occurrence of any adverse events.

The tablets were dispensed in a sachet containing two tablets. One of them was the test or reference medication and the other was a dummy tablet either for test or reference medication and 1 month’s supply was issued. Patient consumed the contents of one sachet (two tablets) once a day, at the same time. Both test and reference medication was given for 120 days.

### Outcome variables

The primary outcome variable was the proportion of patients having new peripheral edema (not present at baseline), not attributable to any concomitant drug, as assessed by clinical assessment of pitting edema at three anatomical points on the body: 7 cm proximal to the midpoint of the medial malleolus, behind medial malleolus and dorsum of the foot. This was compared against baseline, at the end of therapy (120 days).

Antihypertensive efficacy was determined based on decrease in SBP, DBP and mean BP after each month of therapy with the responder rate defined as subjects showing reduction in SBP by ≥20 mmHg and or DBP by ≥10 mmHg or those achieving SBP ≤140 mmHg and DBP ≤90 mmHg at end of therapy.

Secondary outcome variables were, change in the magnitude of pitting edema scores (combined scores for all 3 points), difference in patient assessment questionnaire after each month of therapy, percentage of the subjects experiencing any drug related adverse events and the change in investigational parameters between baseline and after completion of therapy. There were no changes to trial outcomes after the trial commencement***.*** Subjects were evaluated for efficacy and safety on day 30, day 60, day 90 and day 120 of therapy.

### BP measurement and laboratory tests

At each visit, arterial blood pressure was measured using calibrated and validated digital BP apparatus with an appropriate cuff size and a thermal printer (Omron 705CP II upper arm blood pressure monitor with thermal printer), recommended for the use in clinical trials, in accordance with British Hypertension Society published guidelines [22]. At each visit, after the patient had been sitting for 5 min SBP and DBP were measured twice at 2-min intervals. The blood pressure recorded with the thermal printer was taken and pasted on the case record form (CRF).

Laboratory investigations were done in the laboratory of the Department of Pharmacology, Faculty of Medicine, University of Colombo on screening visit and day 120. On the last visit, subject returned the empty sachets and unused medicines***.***

### Assessment of ankle edema

Ankle edema was assessed using clinical assessment and patient questionnaire. The clinical assessment method was done using an adapted technique described for evaluation of edema [23] and the scoring definitions have been changed to identify both the depth of oedema and recovery time of oedema. Pit depth was estimated visually and score recorded and recovery time was given in seconds as also described by Brodovicz et al. Edema assessment was repeated at the three anatomical locations described earlier. The pit depth was estimated visually and a score was given with 0 for no clinical edema, 1 for slight pitting up to 2 mm depth with no visible distortion and increasing in severity up to 4 for a very deep pit of 8 mm with gross distortion of extremity. Pitting edema score was noted as the total score of all 3 points.

Patient-reported edema was recorded using a standardized and validated questionnaire. It included five questions to assess the presence and severity of edema as reported by patients, over the past week with the highest score indicating maximum discomfort.

Adverse events were monitored in every clinic visit. The investigators determined severity, causality and the drug responsible for adverse event. Every adverse event was reported in the CRF and all serious adverse events were reported to the ethics committee and CDDA.

### Sample size

Considering the average incidence of peripheral edema with conventional amlodipine and (S)-Amlodipine to be 16 and 1.56 % respectively from previous studies [6, 20], a sample size of 73 subjects in each group was found to be sufficient to reveal this difference with 80 % power at 5 % significance level. A total of 200 subjects were screened to randomise 172 subjects in to the study to achieve sample size of 146 for analysis after allowing for 15 % dropouts. Interim analysis was not planned nor done and there were no stopping guidelines.

### Randomization procedure

The eligible subjects were randomized to test or reference groups according to the randomization schedule generated by sponsor using online software available at www.randomization.com, in blocks of 10. Randomization chart was available with sponsor and trial site, which was kept in a sealed envelope under lock and key to conceal the allocation and the sealed envelope was checked during monitoring visits. The investigators and research assistants enrolled and assigned interventions in a blinded manner, according to the predetermined allocation sequence.

### Blinding

This was a double-blind, double-dummy study with both the subjects and investigators kept unaware of treatment given. For blinding purposes, both formulations were dispensed along with a dummy tablet identical to the test or reference formulation by appearance, weight and odor. The double-blinding was maintained throughout the study.

### Tests for compliance

Subjects returned the unused medications to the investigators. Manual count of such returns were done and recorded in the CRF. Failure of the patient to come for follow-up was considered as failure of compliance.

### Concomitant treatment

Enrolled subjects continued the BB and ACEI/ARB they were taking prior to randomization. The dosage of these drugs were not altered or modified. Additional antihypertensive drugs were not added to the investigational drug.

### Statistical methods

Intension to treat analysis (ITT) for safety data and per protocol analysis for efficacy data was performed. Mean SBP, DBP and mean BP were calculated as mean ± standard deviation (SD) and compared between the groups/baseline values using *t*-test. Fischer’s exact test was applied to observe if there was significant difference between responder rates and to observe if there were significant difference between proportions of subjects having peripheral edema not attributable to any concomitant drug. Changes in the scores of pitting edema test and scores of patient assessment questionnaire were compared between the groups using Mann-Whitney test. Tolerability was assessed by evaluating the proportion of patients reporting side effects not attributed to any concomitant drug with the global assessment of the subject or physician about the tolerability and efficacy. Fischer’s exact test was applied to observe if there was significant difference between the proportions. Laboratory investigation values were calculated as mean ± SD and compared between the groups or baseline values using *t*-test. For all statistical tests, an overall *p* value of less than or equal to 0.05 was considered as significant. The statistical analysis was performed using Graph Pad InStat 3.00.

## Results

### Participants

**Fig. 1:**
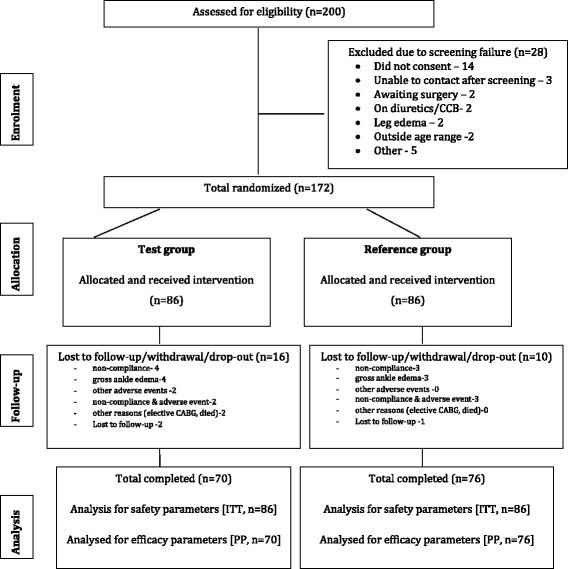
Consort diagram showing the flow of participants

Of the 172 patients randomized, 146 patients (amlodipine, *N =* 76 and S-amlodipine, *N =* 70) completed 120 days of the study duration (Fig. 1).

The baseline characteristics (age, weight, gender, concomitant illness and laboratory values) were comparable between the 2 groups (Table 1). Mean age was 57 years and majority were males in both groups. Although 41 % in the (S)-amlodipine group and 34 % in the racemic amlodipine group had type 2 diabetes mellitus, this difference was not statistically significant.

**Table 1:** Baseline characteristics (for completed patients, *N =* 146)

Parameter	Test group	Reference group	*p*
Number of patients	70	76	na
Age in years; (mean ± SD)	56.46 ± 5.80	56.83 ± 5.57	ns
Weight; (kg) (mean ± SD)	63.82 ± 9.33	65.72 ± 11.94	ns
Male: Female	48:22	52:24	ns
Patients with type II diabetes; %, (n/N)	41.43, (29/70)	34.21, (26/76)	ns
Patients with history of angina; %, (n/N)	5.71, (4/70)	2.63, (2/76)	ns
Patients on captopril; %, (n/N)	32.85 % (23/70)	30.26 % (23/76)	ns
Patients on enalapril; %, (n/N)	31.42 % (22/70)	31.57 % (24/76)	ns
Patients on losartan;%, (n/N)	34.28 % (24/70)	38.15 % (29/76)	ns
Patients on atenolol; %, (n/N)	67.14 % (47/70)	63.15 % (48/76)	ns
Patients on carvedilol; %, (n/N)	14.28 % (10/70)	25 % (19/76)	ns
Patients on metoprolol;%, (n/N)	11.42 % (8/70)	7.89 % (6/76)	ns
Patients on bisoprolol; %, (n/N)	4.28 % (3/70)	2.63 % (2/76)	ns
Hb (mean ± SD)	13.61 ± 1.73	13.57 ± 1.55	ns
Total WBC count (mean ± SD)	7.67 ± 1.95	7.52 ± 1.91	ns
SGOT (mean ± SD)	32.94 ± 13.45	35.41 ± 19.20	ns
SGPT (mean ± SD)	35.86 ± 18.29	34.95 ± 20.79	ns
LDL (mean ± SD)	82.38 ± 26.83	77.26 ± 31.24	ns
HDL (mean ± SD)	40.24 ± 9.18	38.26 ± 8.19	ns
VLDL (mean ± SD)	29.29 ± 11.70	34.13 ± 22.02	ns
Total cholesterol (mean ± SD)	152.17 ± 33.59	150.28 ± 39.42	ns
Triglycerides (mean ± SD)	146.24 ± 58.34	158.25 ± 76.50	ns
Fasting blood glucose (mean ± SD)	116.57 ± 43.35	120.09 ± 52.84	ns
Serum creatinine (mean ± SD)	0.90 ± 0.31	0.87 ± 0.25	ns

*N* total number of patients, *n* number of patients with the variable, *SD* standard deviation, *na* value not assessed, *COPD* chronic obstructive pulmonary disease, *ns* value not statistically significant with *p* > 0.05; Mann-Whitney test was applied for continuous variables and Fisher’s Exact test was applied for categorical variables

The other antihypertensives taken were captopril, bisoprolol, enalapril, atenolol, carvedilol, losartan and metoprolol, with comparable distribution in both groups (Table 1). Adherence to therapy was also equal between the two groups as indicated by mean actual pill count 116.70 ± 3.24 vs 116.41 ± 3.28 and percentage pill count of the ideal count 97.25 ± 2.70 % vs. 97.01 ± 2.74 % in (S)-amlodipine and racemic amlodipine groups respectively. After 90 days, most patients in both groups were on lower dose with significantly more patients in the racemic amlodipine group on the lower strength of the medication (80 % in racemic amlodipine and 61 % in (S)-amlodipine; *p* = 0.0098).

### Incidence of edema

The incidence of new edema any time after randomization, was 31.40 % for test and 46.51 % for reference (*p* = 0.0301); The absolute risk reduction (ARR) of new edema was 15.1 % with relative risk reduction (RRR) of 32.47 % and number needed to treat (NNT) of seven. In 11 out of 27 (55 %) patients in S-amlodipine group the edema resolved during the study, while edema resolution was 17/40 (43.59 %) in racemic amlodipine group (*p* = ns).

After 120 days of therapy, pitting edema score increased significantly in racemic amlodipine group compared to (S)-amlodipine (*p* = 0.038) and increment of score was highly significant (*p* < 0.0001) with amlodipine compared with baseline value (Table 2). The temporal relationship of edema indicates that most patients had highest edema scores at 30 days in V1 in both groups but edema scores reduced over the next 3 months (Table 2). Patient assessment questionnaire for edema score also followed somewhat similar pattern (Table 3).

**Table 2:** Evaluation of pitting edema score (ITT analysis, *N =* 172)

Variable	Test group		Reference group		p*
	N	Pitting edema score (mean ± sd)	N	Pitting edema score (mean ± sd)	
Baseline (day 0), V1	86	00.00 ± 00.00	86	0.05 ± 00.34	na
After 30 days of therapy, V2	86	01.09 ± 02.47	86	00.97 ± 01.88	ns
After 60 days of therapy, V3	86	00.44 ± 01.36	86	00.88 ± 01.88	ns
After 90 days of therapy, V4	86	00.38 ± 01.21	86	00.73 ± 01.66	ns
After 120 days of therapy, V5	86	0.33 ± 01.00	86	00.83 ± 01.52	0.0380
p**		na		<0.0001	

*ITT* intention to treat, *N* number of patients, *V1-V5* Visits 1–5, *na* not applicable, *ns* not significant

Mann-Whitney test was applied; *p value, when compared between the groups; **p value, when V5 value compared with baseline value in same group; *p* <0.05 = statistically significant

**Table 3:** Evaluation of patient assessment questionnaire for edema (ITT analysis, *N =* 172)

Variable	Test group		Reference group		p*
	N	Patient questionnaire score (mean ± sd)	N	Patient questionnaire score (mean ± sd)	
Baseline (day 0), V1	86	00.00 ± 00.00	86	00.14 ± 01.02	na
After 30 days of therapy, V2	86	02.34 ± 05.23	86	02.07 ± 04.31	ns
After 60 days of therapy, V3	86	01.03 ± 03.34	86	02.29 ± 04.60	ns
After 90 days of therapy, V4	86	01.06 ± 03.41	86	01.81 ± 04.02	ns
After 120 days of therapy, V5	86	01.11 ± 03.35	86	02.60 ± 04.50	0.0363
p**		na		<0.0001	

*ITT* intention to treat, *N* number of patients, *V1-V5* visits 1–5, *na* not applicable, *ns* not significant

Mann-Whitney test was applied; *p value, when compared between the groups; **p value, when V5 value compared with baseline value in same group; *p* <0.05 = statistically significant

In the racemic amlodipine group there were 26 males (age: 57.42 ± 05.57 years), 14 females (age:58.57 ± 04.18 years) and in the (S)-amlodipine group 14 males (age: 57.43 ± 04.67 years), 13 females (age: 57.46 ± 06.05 years) who developed edema. There was no significant difference in the incidence of edema between males and females in either group.

The mean age of patients having edema in test group (*N =* 27) was 57.44 ± 05.27 and in reference group (*N =* 40) was 57.83 ± 05.24. The mean age of patients not having edema in test group (*N =* 59) was 56.10 ± 05.88 and in reference group (*N =* 46) was 56.11 ± 05.57. There was no significant difference in the age of patients with edema or without edema in each group.

### Blood pressure control

The SBP and DBP and the mean BP decreased significantly in both groups, without any significant difference between groups by day 120 (Table 4). The lowest mean blood pressure was recorded at 60 days in V3 and although the BP has increased slightly over the next 2 visits, the mean BP was significantly lower by the end of the study in both arms (Table 4). The percentage of responders was 98.57 % in test and 98.68 % in reference group (per protocol analysis, *N =* 146, *p* = ns; Table 5). There was no difference in heart rate between the two groups over time.

**Table 4:** Evaluation of mean blood pressure at baseline and after therapy (PP analysis; *N =* 146)

Variable	Test group		Reference group		*p* *
	N	mm of Hg (mean ± SD)	N	mm of Hg (mean ± SD)	
Baseline (day 0), V1	70	113.01 ± 25.93	76	113.99 ± 12.17	ns
After 30 days of therapy, V2	70	101.08 ± 17.92	76	95.33 ± 12.34	ns
After 60 days of therapy, V3	70	90.08 ± 06.01	76	89.81 ± 8.02	ns
After 90 days of therapy, V4	70	102.50 ± 14.34	76	99.01 ± 08.45	ns
After 120 days of therapy, V5	70	94.42 ± 17.25	76	95.89 ± 03.34	ns
p**		<0.0001		<0.0001	

*PP* per protocol, *N* number of patients, *SD* standard deviation, *ns* not significant

Paired *t* test was applied; *p value, when compared between the groups; **p value, when V5 value compared with baseline value in same group; *p* <0.05 = statistically significant

**Table 5:** Percentages of blood pressure responders at 120 days (PP analysis; *N =* 146)

Drug	Responders	*p*
Test group %, (n/N)	98.57, (69/70)	ns
Reference group %, (N)	98.68, (75/76)	

*N* total number of patients, *n* number of responders, Fisher’s Exact test was applied; *p* <0.05 = statistically significant, *ns* not significant, *PP* per protocol

*Responders:* Subjects showing reduction in systolic blood pressure by ≥20 mmHg and / or diastolic blood pressure by ≥10 mm or those achieving systolic blood pressure ≤140 mmHg and diastolic blood pressure ≤90 mm of Hg

### Safety

The global assessment for efficacy and tolerability to treatment was similar in both the groups. The adverse events with ITT analysis,(*N =* 172; Table 6) showed that the most common adverse events were pitting edema and increased urinary frequency (24.41 % in each group). Other adverse events were headache, muscle cramps, constipation, peri-orbital edema, vertigo, dizziness, and rash. No difference was observed in adverse events in the two groups. There were two serious adverse events (SAE) reported. One death occurred in a patient randomized to test group after 15 days of randomization; the cause of death was acute myocardial infarction, and this SAE was considered unrelated to the study drug. A patient who was hospitalized due to vasculitic rash on lower limbs in reference group was the other SAE. This was also considered unrelated to the study treatment.

**Table 6:** Adverse events (Intention to treat analysis, *N =* 172)

Adverse events	Test group %, (n/N)	Reference group %, (n/N)	*P*
Pitting edema	31.40, (27/86)	46.51, (40/86)	0.0301
Increased urinary frequency	24.41, (21/86)	24.41, (21/86)	ns
Muscle cramps	4.65, (4/86)	2.32, (2/86)	ns
Rash on face/arms	2.32, (2/86)	0 (0/86)	ns
Headache	2.32, (2/86)	3.48, (3/86)	ns
Constipation	1.16, (1/86)	2.32 (2/86)	ns
Peri-orbital edema	1.16, (1/86)	0 (0/86)	ns
Vertigo & dizziness	0 (0/86)	1.16, (1/86)	ns
Vasculitic rash on lower limbs	0 (0/86)	1.16, (1/86)	ns
Death	1.16, (1/86)	0 (0/86)	ns
Total no. of AEs	68.60, (59/86)	81.39, (70/86)	0.03

*AE* adverse event, *N* total number of patients, *n* number of AEs, Fisher’s exact test was applied. *p* < 0.05 = statistically significant, *ns* value not significant

There were no clinically significant differences in the biochemical parameters, urinary and electrocardiographic tests before and after treatments.

## Discussion

This is probably the first adequately powered randomised controlled clinical trial, evaluating leg edema as a primary outcome which recruited both men and women, comparing efficacy and safety of (S)-amlodipine vs racemic amlodipine. CCBs are associated with a considerable risk of peripheral oedema [5, 6, 13, 24, 25], that may reduce patient compliance or necessitate switching to a different drug [26]. This study shows that significantly high percentage (46.5 %) of patients developed peripheral edema while on racemic amlodipine compared to the percentage developing edema with (S)-amlodipine (31.4 %). The difference in the two groups is significant and represents a RRR of approximately 33 % with a NNT of only seven.

These results are in contrast to a systematic review and meta analysis of the clinical trials comparing (*S*)-amlodipine and racemic amlodipine [17] which did not show any significant difference in incidence of edema when only high-quality trials were included, although when all the trials were considered, the edema incidence was significantly less with (S)-amlodipine compared to racemic amlodipine.

The edema incidence in our trial was much higher than most of the previously reported incidence with racemic amlodipine at the doses used, varying from 2 to 32 % [5, 6, 18, 24]. The main reason for this high incidence is probably active surveillance for edema in this trial both by patient assessment questionnaire for edema and objective assessment by investigators using an edema score. It is noted that considerably higher rates of adverse events are reported by active surveillance systems when compared with passive systems [27]. Rates of edema reported in clinical trials involving CCBs may have been affected by active or passive surveillance for edema, which could have given the wide variation in the reported edema incidence in the patients given CCB [28–30].

Higher edema scores by patients is likely to result in higher degree of discomfort for many patients [31]. Therefore in populations that experience a high incidence of edema with racemic amlodipine, use of chirally pure (S)-amlodipine would be advantageous due to lower incidence of edema which could result in improved adherence to therapy and hence blood pressure control.

The cause of CCB-induced edema is elevated capillary hydrostatic pressure. This results from preferential dilation of pre-capillary vessels. Renin-angiotensin system (RAS) blockers such as ACEIs and ARBs normalize hydrostatic pressure by causing post-capillary dilation. Therefore they could be used for prevention or reversal of CCB-induced edema [11].

Several clinical trials have shown that the incidence of oedema is lower in patients who receive ACEI/CCB or ARB/CCB combination therapy when compared to those treated with CCB therapy alone. [17, 28, 32] A meta-analysis that included 82 studies that compared the safety and efficacy of ACEI/CCB therapy against nine monotherapy regimens stated that this drug combination was associated with a lower rate of side effects [33]. It was also noted that the side effects led to withdrawal of amlodipine or nifedipine monotherapy [33]. In our study as both arms received beta-blockers and ACE-I/ARBs, the contribution of these medications to edema can be assumed to be equal in both the groups. The incidence of leg edema could have been higher in both groups if the patients were not on concomitant ACEI/ARBs. As the cause of oedema is pre capillary vasodilatation, higher rates of oedema associated with CCBs may be occurring more in tropical countries due to the warm environment resulting in vasodilatation in peripheries. The reported rates of edema with CCBs seem to be higher in tropical countries with warm environments such as India and Brazil [19, 21, 34] while the rates seem lower in countries with average lower temperatures such as USA and Korea [18, 28]. Although there are no studies supporting this theory in literature, seasonal variation in incidence of edema has been reported [5] and authors have observed patients reporting resolution of edema on visiting cold climates and reappearance of edema when returning to warm environments.

In this trial both arms received triple therapy with a CCB, ACEI/ARB and a BB and excellent blood pressure control was achieved in 98 % of patients. Multiple trials have previously reported that there are highly significant cardiovascular outcome benefits of antihypertensives given in combination [35] and the use of combination therapy in our study population is consistent with the recommendations of Eighth Report of the Joint National Committee on Prevention, Detection, Evaluation, and Treatment of High Blood Pressure (JNC8) [36]. Currently amlodipine is one of the most efficacious drugs recommended for triple therapy in uncontrolled hypertension [37], and this study also confirms that. We observed that most patients needed only a low dose of amlodipine to control the blood pressure in each arm with triple therapy with the percentage of patients down titrated to low dose significantly higher in the racemic amlodipine group (80 %) compared to the percentage in (S)-amlodipine group (61 %). The temporal relationship of edema showed higher edema scores at 30 and 60 days and resolution thereafter in both groups but mainly in (S)-amlodipine group over the next 2–3 months (Tables 2 and 3). This could probably be explained by the higher doses used initially in this trial in both arms with subsequent down titration of doses. It is well known that edema with CCB occurs in a dose dependent manner [5]. Other reasons such as postural effects due to the advice given to patients when they developed edema may also have contributed to resolution of edema. Edema score increasing initially with subsequent resolution is also supported by lowest mean BP recorded at 60 days (Table 4) with subsequent slight increment of BP noted, which again probably occurred due to down titration of drug doses.

Adverse events, in particular leg edema with amlodipine has been reported to occur more in female patients in some studies [1, 6, 38]. Another study among female Korean patients with mild to moderate hypertension, quantified pedal edema during treatment with (S)-Amlodipine nicotinate versus amlodipine besylate [38] and concluded that females had a reduced level of ankle edema, with no significant difference in antihypertensive efficacy. Thus it is important to note that our study did not show any significant difference in incidence of edema between males and females. There was also no significant difference between the ages of the patients who developed edema compared to those who did not develop edema in both groups although some studies report edema occurrence with advancing age [5].

Notable amongst the other adverse events, was the high incidence of frequent urination, noted in almost 25 % in both the groups. Amlodipine is known to have natriuretic effect [39] although reported frequency is less than 1 % according to the prescribing information for amlodipine besylate tablets. However there are previous studies reporting higher incidence of increased urinary frequency [40].

The natriuretic effect of CCBs augments the antihypertensive effect of ACEIs [41, 42] and this better antihypertensive efficacy in combination is another advantage cited for combining CCBs with RAS inhibitors such as ACEI/ARBs in addition to mitigating edema due to CCB [13, 34].

Muscle cramps, headache and constipation were reported by 1–5 % of patients in both groups which are well recognised side effects of CCBs and incidence of these side effects were not significantly different in both groups and from previous reports.

## Conclusion

The current recommendation in most hypertension treatment guidelines [43], is combination antihypertensive therapy for patients whose BP is 20 mm Hg above the systolic goal or 10 mmHg above the diastolic goal, which were the inclusion criteria for BP this clinical trial. This study which randomised patients with inadequately controlled BP on ACEI/ARB and BB to compare the efficacy and safety of (S)-amlodipine and racemic amlodipine, with new onset leg edema as a primary outcome, showed significantly reduced incidence of edema with (S)-amlodipine compared to racemic amlodipine with excellent blood pressure control in both arms. As development of edema with CCB results in poor adherence, poor BP control and necessitate switching therapy, using (S)-amlodipine is likely to result in better tolerance and BP control. This is particularly so when used in combination therapy for control of hypertension, especially in populations that experience high rates of edema with CCB.

## Abbreviations

ACEI: Angiotensin converting enzyme inhibitors
AE: Adverse events
ARB: Angiotensin receptor blockers
ARR: Absolute risk reduction
BB: Beta-blockers
BP: Blood pressure
CABG: Coronary artery bypass grafting
CCB: Calcium channel blocker
CDDA: Cosmetics devices and drugs authority
CIS: Commonwealth of independent States
CRF: Case record form
DBP: Diastolic blood pressure
ECG: Electrocardiogram
GCP: Good Clinical Practice
ITT: Intention to treat
JNC8: Eighth report of the joint national committee
NNT: Number needed to treat
OPD: Out patient department
PTCA: Percutaneous trans coronary angiogram
RAS: Renin-angiotensin system
RRR: Relative risk reduction
SAE: Serious adverse events
SBP: Systolic blood pressure
SD: Standard deviation
TIA: Transient ischemic attack
US: United States
WHO: World health organization
